# Hospital mortality of patients aged 80 and older after surgical repair for type A acute aortic dissection in Japan

**DOI:** 10.1097/MD.0000000000004408

**Published:** 2016-08-07

**Authors:** Tetsu Ohnuma, Daisuke Shinjo, Kiyohide Fushimi

**Affiliations:** Department of Health Policy and Informatics, Tokyo Medical and Dental University Graduate School, Tokyo, Japan.

**Keywords:** aorta, cardiovascular diseases, mortality, surgery

## Abstract

To evaluate whether patients aged 80 and older have higher risk of hospital mortality after repair of type A acute aortic dissection (TAAAD).

Emergency surgery for TAAAD in patients aged 80 and older remains a controversial issue because of its high surgical risk.

Data from patients who underwent surgical repair of TAAAD between April 2011 and March 2013 were retrospectively extracted from the Japanese Diagnosis Procedure Combination database. The effect of age on hospital mortality was evaluated using multivariate logistic regression analysis.

A total of 5175 patients were enrolled. The mean age of patients was 67.1 ± 13.0 years, and the male:female ratio was 51:49. Patients aged 80 and older more frequently received tracheostomy than their younger counterparts (9.5% vs 5.4%, *P* <0.001). Intensive care unit and hospital stays were significantly longer in the elderly cohort versus the younger cohort (7.6 vs 6.7 days, *P* <0.001, and 42.2 vs 35.8 days, *P* <0.001, respectively). Logistic regression analysis showed that age ≥80 years was significantly associated with a higher risk of hospital mortality (adjusted odds ratio, 1.62; 95% confidence interval, 1.28–2.06; *P* <0.001). In linear regression analysis, age ≥80 years was also significantly associated with longer hospital stay (*P* = 0.007).

In a large, nationwide, Japanese database, patients aged 80 and older were at increased risk of hospital mortality and length of hospital stay.

## 1 Introduction

Type A acute aortic dissection (TAAAD) is a cardiovascular emergency associated with high mortality and morbidity. Despite continuing improvements in surgical and medical management, TAAAD-associated hospital mortality has been reported to range from 4.1% to 32.5%.^[1–3]^

Urgent surgery for TAAAD in patients aged 80 and older remains a controversial issue. If the surgical treatment decision is avoided or withdrawn, approximately 50% of patients die within 48 hours.^[4]^ A systematic review and meta-analysis that included studies on surgical repair of TAAAD in patients aged 80 and older showed that after surgery for TAAAD, the immediate mortality rate for such patients varied from 3.7% to 83%, although the number of patients in each study was small and the studies that showed higher mortality rate were older and do not reflect advances in surgical techniques.^[5]^ Moreover, less invasive procedures for TAAAD, such as thoracic endovascular aortic repair (TEVAR) and transcatheter aortic valve implantation, are available but are still being developed.^[6]^

Therefore, we conducted this study to evaluate whether patients aged 80 and older after repair of TAAAD have increased risk of hospital mortality.

## 2 Methods

### 2.1 Data source

We used the Japanese Diagnosis Procedure Combination (DPC) database, which is a Japanese case-mix classification system linked with a payment system. Details of the DPC were described elsewhere.^[7,8]^ In short, by 2011 more than 1400 acute care hospitals were participating in the DPC database, covering approximately 50% of patients discharged from all Japanese hospitals. The database includes baseline patient information, diagnosis information using the International Classification of Diseases and Injuries 10th revision (ICD-10), medical procedures, medications, and materials.

### 2.2 Study population

The Tokyo Medical and Dental University ethics committee approved this study, and the requirement for informed consent was waived. Data from patients with TAAAD after surgical repair as the principal disease or the disease associated with the highest medical costs between April 2011 and March 2013, were detected by ICD-10 of I71.0 and surgical procedure codes of the Japanese claims classification and retrospectively extracted from the DPC database. Exclusion criteria were as follows: chronic aortic dissection, planned admission, outlier hospital length of stay, and missing data. The final cohort was divided into 2 groups: ≥80 years old and <80 years old.

### 2.3 Variables

Preoperative and intraoperative variables evaluated included age, gender, smoking, Charlson comorbidity index (CCI),^[9]^ hypertension, diabetes, chronic obstructive pulmonary disease (COPD), Marfan syndrome, previous myocardial infarction (MI), chronic kidney disease, malignancy, previous cardiac surgery, and type of surgery (aortic valve replacement, total arch replacement, David procedure, Bentall procedure, and coronary bypass artery graft [CABG]). Postoperative events and outcomes included: reoperation, renal replacement therapy (RRT), length of ventilation, tracheostomy, intensive care unit (ICU) length of stay, hospital length of stay, 30-day mortality, and hospital mortality. Consciousness level was assessed at admission and discharge using the Japan Coma Scale score: 0 (alert), 1–3 (delirious), 10–30 (somnolent), and 100–300 (comatose).^[10]^

### 2.4 Statistical analysis

Data were presented as mean ± standard deviation, or percentages, as appropriate. Either the *χ*^2^ or Fisher exact test was used for nominal variables, and unpaired *t* tests were used to compare continuous variables. A *P* value of <0.05 was considered statistically significant. We did not impute any missing data in the present study.

To identify whether age (<80 vs ≥80 years) was associated with hospital mortality, multivariate logistic regression analysis was performed using the following covariables: gender, smoking, CCI, hypertension, diabetes, COPD, previous MI, chronic kidney disease, malignancy, previous cardiac surgery, consciousness level at admission, aortic valve replacement, total arch replacement, Bentall procedure, David procedure, CABG, reoperation, length of ventilation, tracheostomy, and RRT. No collinearity was found among these variables. A similar multivariate logistic regression analysis was performed to identify whether age was similarly associated with hospital mortality among men and women in the sensitivity analysis. The influence of age and other factors on length of hospital stay were evaluated using linear regression. All analyses were carried out using R statistical software, version 3.2.3 (R Foundation for Statistical Computing, Vienna, Austria).

## 3 Results

**Figure 1:**
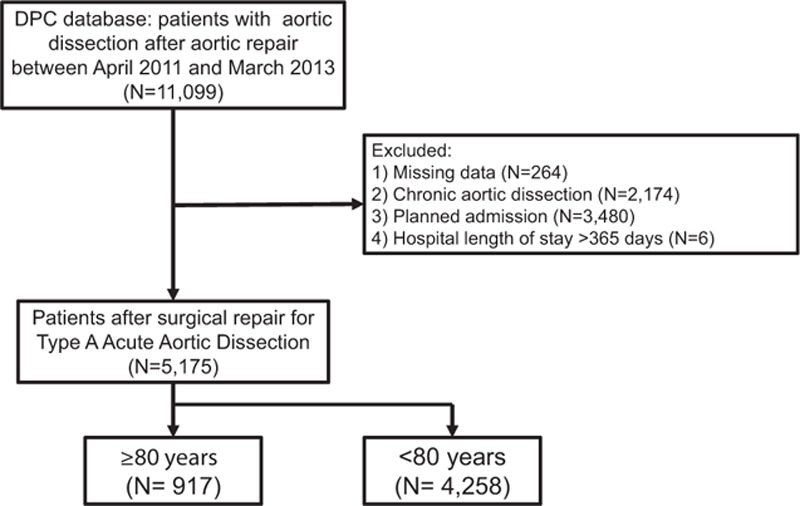
Selection of patients after surgical repair for type A acute aortic dissection.

A total of 5175 patients who underwent surgical repair of TAAAD in 356 hospitals were enrolled between April 2011 and March 2013. As shown in Figure 1, 5660 patients were excluded from the study because of missing data (N = 264), chronic aortic dissection (N = 2174), planned admission (N = 3480), and length of hospital stay over 365 days (N = 6).

Baseline characteristics and surgical procedures are shown in Table 1. The mean age of patients was 67.1 ± 13.0 years, and the male:female ratio was 51:49. Preoperative comorbidities included hypertension (61%), diabetes mellitus (9.7%), and COPD (3.2%), and the mean of the CCI was 0.79 ± 0.91. Consciousness level at admission did not differ between 2 groups: alert (79.1%), delirious (10.6%), somnolent (3.5%), and coma (6.6%). Surgical procedures performed included aortic valve replacement (8.6%), hemi or total arch replacement (41.7%), Bentall procedure (2.4%), David procedure (0.5%), and CABG (4.4%).

**Table 1:**
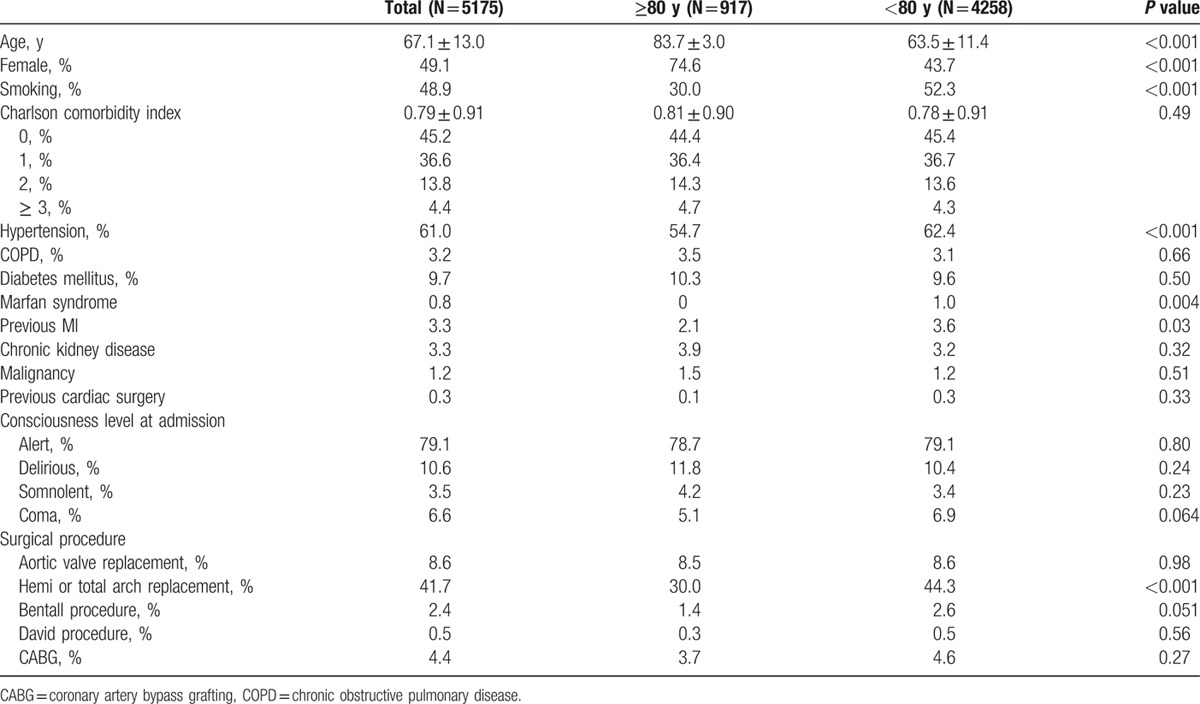
Characteristics of patients with type A acute aortic dissection.

**Figure 2:**
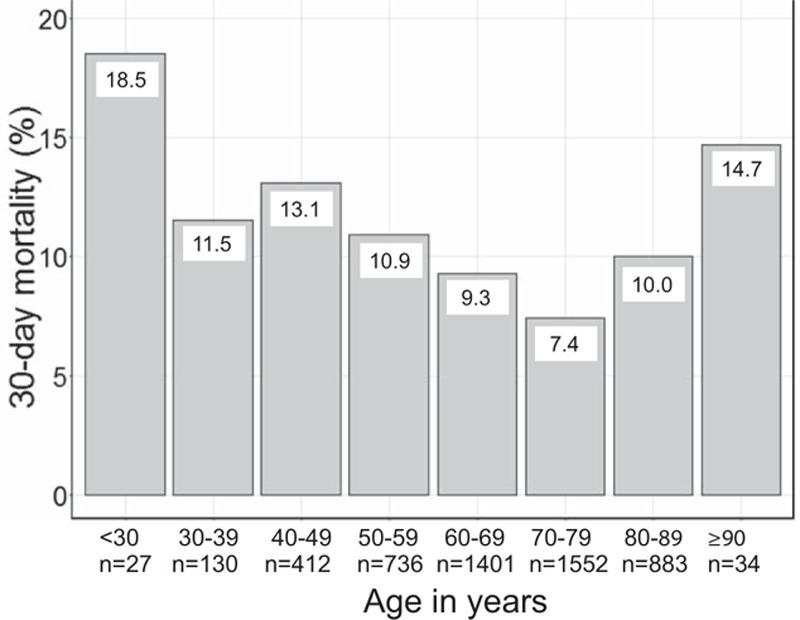
Hospital mortality categorized by age group (10-year increments). The number of patients in each group is shown below the x-axis.

Table 2 shows postoperative complications and outcomes. Approximately 6.3% of patients in each group underwent reoperation. Patients aged 80 and older more frequently received tracheostomy than did those younger than 80 years (9.5% vs 5.4%, *P* <0.001). Mean ventilation days (7.6 ± 16.3 days), renal replacement therapy (6.1%), and consciousness level at discharge did not differ between 2 groups. ICU and hospital stays were significantly longer in the older cohort (7.6 days vs 6.7 days, *P* <0.001, and 42.2 vs 35.8 days, *P* <0.001, respectively). Figure 2 shows that when patients were stratified by age, those older than 90 years had the highest mortality (20.6%).

**Table 2:**
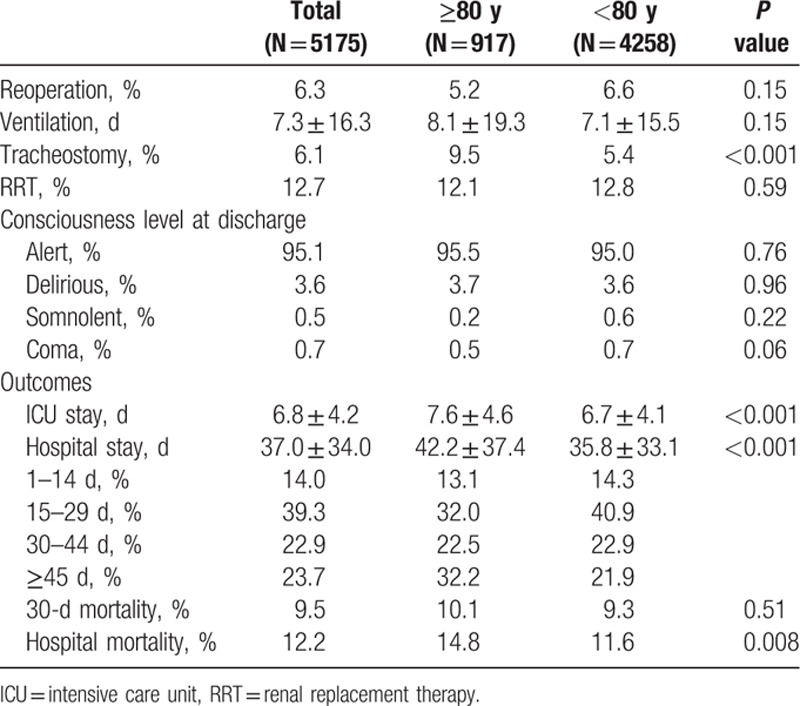
Postoperative outcomes of patients with type A acute aortic dissection.

In the logistic regression analysis, with hospital mortality as the dependent variable and adjusting for confounders, age ≥80 years was significantly associated with higher risk of hospital mortality (adjusted odds ratio, 1.62; 95% confidence interval, 1.28–2.06; *P* <0.001). Female, previous MI, renal replacement therapy, CABG, and Bentall procedure were also significantly associated with higher hospital mortality (Table 3). In the sensitivity analysis when the cohort was divided by gender, only the group of female patients aged ≥80 years was significantly associated with a higher risk of hospital mortality (adjusted odds ratio, 2.11; 95% confidence interval, 1.55–2.86; *P* <0.001), compared with the male patients (adjusted odds ratio, 1.04; 95% confidence interval, 0.67–1.57; *P* = 0.83). Furthermore, in the linear regression analysis, age ≥80 years was also significantly associated with longer hospital stay (*P* = 0.007).

**Table 3:**
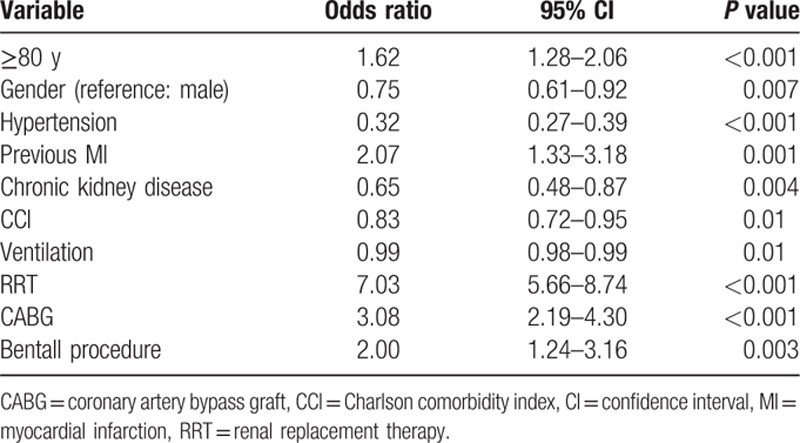
Logistic regression analysis for hospital mortality.

## 4 Discussion

### 4.1 Key findings

In a large database of acute care hospitals in Japan, we found that the hospital mortality rate was higher for patients aged 80 and older (14.8%) than it was for patients younger than 80 years (11.6%). Older age (≥80 years) was associated with higher hospital mortality and longer hospital stay, and this result was especially remarkable among female patients. The mean hospital stay was 6.4 days longer in the elderly group than it was in younger patients.

### 4.2 Relationship to previous studies

Until recently, surgery of the ascending aorta and the aortic arch for aortic dissections carried high mortality and morbidity for elderly patients. A study conducted by Neri et al^[11]^ in 2001 reported that the hospital mortality of 24 octogenarians who underwent repair for TAAAD was 83%. Chavanon et al^[12]^ demonstrated 56.3% hospital mortality in 16 octogenarians who underwent immediate repair of TAAAD. Conversely, an increasing number of elderly patients undergo surgery for TAAAD and achieve acceptable outcomes because of advances in surgical and medical management.^[13]^ In fact, several studies have reported more favorable operative mortalities in elderly patients with TAAAD, ranging between 4.8% and 34.9%.^[6,14–17]^ In our study, hospital mortality was 14.8%, indicating that surgical repair for TAAAD can be performed with a relatively low mortality rate in patients aged 80 years and older.

Nevertheless, the lack of evidence on the benefits of immediate surgical intervention for elderly patients makes the clinical decision difficult. In a 2010 report of the International Registry of Acute Aortic Dissection study, Trimarchi et al^[18]^ reported that age 70 or older was an independent predictor for in-hospital mortality in 936 patients with TAAAD (OR 1.728, 95% CI 1.06–2.816; *P* = 0.0281). Rylski et al^[17]^ showed that octogenarians had significantly higher 30-day mortality rates than did septuagenarians (OR 3.23, 95% CI 1.81–5.72, *P* <0.001), in a multicenter German Registry for Acute Aortic Dissection Type A study from 2006 to 2009 that included 464 patients with TAAAD. Moreover, a systematic review and meta-analysis of 6 studies from 2001 to 2011 that was conducted by Biancari et al^[5]^ demonstrated that patients aged 80 and older had a significantly higher risk of operative mortality than did younger patients (risk ratio 2.32, 95% CI 1.47–3.66, *P* <0.001). In line with previous investigations, the present, large study revealed that being age 80 and older was an independent risk factor for hospital mortality among patients with TAAAD. Although several studies showed immediate operative mortality rates after surgical repair for TAAAD were similar between elderly and young patients, many of those studies were retrospective analyses in single institutions with just small cohorts of elderly patients.^[5–7]^

Several possible factors may explain why hospital mortality was higher in patients aged 80 and older with TAAAD. First, elderly patients tend to show fewer symptoms, such as acute onset of pain.^[19]^ Second, more perioperative complications are observed: higher frequency of cardiac tamponade, prolonged intubation, supra-aortic branch vessel involvement, and dissections extending to the abdominal aorta and iliac arteries.^[17]^ Lastly, neurological complications due to stroke are more frequent in older patients after surgical treatment of TAAAD.^[20]^ Those facts may lead to more serious conditions for such patients, resulting in poorer outcomes.

### 4.3 Significance and implications

On the basis of our findings, patients aged 80 and older who undergo surgical repair for TAAAD have a lower rate of hospital mortality (14.8%) than do patients of similar age with TAAAD who do not undergo surgery (59%),^[5]^ indicating that compared with medical management, surgical treatment of such patients may be reasonable. However, because the results of our study imply that there are few benefits of surgical repair for older patients, especially for women, the clinical indication of the operation should be carefully decided on a case-by-case basis.

Alternatively, several studies have reported the feasibility and success of TEVAR, which is less invasive, to reduce postoperative complications.^[21–23]^ This approach may be beneficial for elderly patients. However, further studies are required to confirm its efficacy, because only a few small studies have evaluated its outcomes and the technique has various limitations because of anatomical complexities in the ascending aorta and aortic arch.^[22]^

### 4.4 Strengths and limitations

This study has major strengths: it is the largest reported investigation of its kind (in terms of patient number) and is derived from a national administrative database in Japan.

The present study also has several limitations. First, the main limitation is that our database did not include severely ill patients who did not undergo surgery for TAAAD or who died on the way to hospital. Second, quality of life and long-term outcomes were unavailable in this retrospective observational study; such parameters are of critical concern, especially among elderly patients who may suffer from declined quality of life after cardiac surgery.^[24]^ Third, some variables that could be considered as unmeasured confounders were not included in our study, such as preoperative symptoms, laboratory data, time of surgery, other postoperative complications such as sepsis and stroke, and individual surgeon experience. Finally, data on quality of care by medical professionals in the intensive care unit that contributed to the care of each patient were lacking.

## 5 Conclusion

In a large national database, patients aged 80 and older were at increased adjusted odds of hospital mortality and increased length of hospital stay following emergency surgery for TAAAD. Further studies assessing the burden of treatments, long-term survival, and quality of life will be required to establish the role of emergency surgery for this cohort.

## References

[1] Sadi L T⊘nnessen T Pillgram-Larsen J Short and long-term survival in type A aortic dissection justifies the operative risk and effort. *Scand Cardiovasc J* 2012; 46:45–50. 22029877 10.3109/14017431.2011.626439

[2] Ohnuma T Kimura N Sasabuchi Y Lower heart rate in the early postoperative period does not correlate with long-term outcomes after repair of type A acute aortic dissection. *Heart Vessels* 2015; 30:355–361. 24566590 10.1007/s00380-014-0486-7 PMC4427614

[3] Santini F Montalbano G Messina A Survival and quality of life after repair of acute type A aortic dissection in patients aged 75 years and older justify intervention. *Eur J Cardiothorac Surg* 2006; 29:386–391. 16434205 10.1016/j.ejcts.2005.12.016

[4] Rylski B Hoffmann I Beyersdorf F Multicenter Prospective Observational Study. Acute aortic dissection type A: age-related management and outcomes reported in the German Registry for Acute Aortic Dissection Type A (GERAADA) of over 2000 patients. *Ann Surg* 2014; 259:598–604. 23657079 10.1097/SLA.0b013e3182902cca

[5] Biancari F Vasques F Benenati V Contemporary results after surgical repair of type A aortic dissection in patients aged 80 years and older: a systematic review and meta-analysis. *Eur J Cardiothorac Surg* 2011; 40:1058–1063. 21561787 10.1016/j.ejcts.2011.03.044

[6] El-Sayed Ahmad A Papadopoulos N Detho F Surgical repair for acute type A aortic dissection in octogenarians. *Ann Thorac Surg* 2015; 99:547–551. 25476805 10.1016/j.athoracsur.2014.08.020

[7] Matsuda S Ishikawa KB Kuwabara K Development and use of the Japanese case-mix system. *Eurohealth* 2008; 14:25–30.

[8] Shinjo D Fushimi K Preoperative factors affecting cost and length of stay for isolated off-pump coronary artery bypass grafting: hierarchical linear model analysis. *BMJ Open* 2015; 5:e008750. 10.1136/bmjopen-2015-008750 PMC4654398 26576810

[9] Sundararajan V Quan H Halfon P International Methodology Consortium for Coded Health Information (IMECCHI). Cross-national comparative performance of three versions of the ICD-10 Charlson index. *Med Care* 2007; 45:1210–1215. 18007172 10.1097/MLR.0b013e3181484347

[10] Shigematsu K Nakano H Watanabe Y The eye response test alone is sufficient to predict stroke outcome—reintroduction of Japan Coma Scale: a cohort study. *BMJ Open* 2013; 3:e002736. 10.1136/bmjopen-2013-002736 PMC3641437 23633419

[11] Neri E Toscano T Massetti M Operation for acute type A aortic dissection in octogenarians: is it justified? *J Thorac Cardiovasc Surg* 2001; 121:259–267. 11174731 10.1067/mtc.2001.112205

[12] Chavanon O Costache V Bach V Preoperative predictive factors for mortality in acute type A aortic dissection: an institutional report on 217 consecutives cases. *Interact Cardiovasc Thorac Surg* 2007; 6:43–46. 17669765 10.1510/icvts.2006.131433

[13] Tsai TT Trimarchi S Nienaber CA Acute aortic dissection: perspectives from the International Registry of Acute Aortic Dissection (IRAD). *Eur J Endovasc Surg* 2009; 37:149–159. 10.1016/j.ejvs.2008.11.032 19097813

[14] Matsushita A Tabata M Fukui T Outcomes of contemporary emergency open surgery for type A acute aortic dissection in elderly patients. *J Thorac Cardiovasc Surg* 2014; 147:290–294. 23228401 10.1016/j.jtcvs.2012.11.007

[15] Komatsu K Takano T Terasaki T Surgical outcomes of acute type A aortic dissection in elderly patients. *Ann Thorac Surg* 2014; 97:1576–1581. 24636709 10.1016/j.athoracsur.2014.01.045

[16] Kilic A Tang R Whitson BA Outcomes in the current surgical era following operative repair of acute Type A aortic dissection in the elderly: a single-institutional experience. *Interact Cardiovasc Thorac Surg* 2013; 17:104–109. 23563053 10.1093/icvts/ivt155 PMC3686405

[17] Rylski B Suedkamp M Beyersdorf F Outcome after surgery for acute aortic dissection type A in patients over 70 years: data analysis from the German Registry for Acute Aortic Dissection Type A (GERAADA). *Eur J Cardiothorac Surg* 2011; 40:435–440. 21247773 10.1016/j.ejcts.2010.11.073

[18] Trimarchi S Eagle KA Nienaber CA International Registry of Acute Aortic Dissection Investigators. Role of age in acute type A aortic dissection outcome: report from the International Registry of Acute Aortic Dissection (IRAD). *J Thorac Cardiovasc Surg* 2010; 140:784–789. 20176372 10.1016/j.jtcvs.2009.11.014

[19] Hiratzka LF Bakris GL Beckman JA American College of Cardiology Foundation/American Heart Association Task Force on Practice Guidelines, American Association for Thoracic Surgery, American College of Radiology, American Stroke Association, Society of Cardiovascular Anesthesiologists, Society for Cardiovascular Angiography, Interventions, Society of Interventional Radiology, Society of Thoracic Surgeons, Society for Vascular Medicine. 2010 ACCF/AHA/AATS/ACR/ASA/SCA/SCAI/SIR/STS/SVM Guidelines for the diagnosis and management of patients with thoracic aortic disease. A Report of the American College of Cardiology Foundation/American Heart Association Task Force on Practice Guidelines, American Association for Thoracic Surgery, American College of Radiology, American Stroke Association, Society of Cardiovascular Anesthesiologists, Society for Cardiovascular Angiography and Interventions, Society of Interventional Radiology, Society of Thoracic Surgeons, and Society for Vascular Medicine. *J Am Coll Cardiol* 2010; 55:e27–e129. 20359588 10.1016/j.jacc.2010.02.015

[20] Bossone E Corteville DC Harris KM Stroke and outcomes in patients with acute type A aortic dissection. *Circulation* 2013; 128:S175–S179. 24030403 10.1161/CIRCULATIONAHA.112.000327

[21] Sobocinski J O’Brien N Maurel B Endovascular approaches to acute aortic type A dissection: a CT-based feasibility study. *Eur J Vasc Endovasc Surg* 2011; 42:442–447. 21764338 10.1016/j.ejvs.2011.04.037

[22] Kolvenbach RR Karmeli R Pinter LS Endovascular management of ascending aortic pathology. *J Vasc Surg* 2011; 53:1431–1437. 21276685 10.1016/j.jvs.2010.10.133

[23] Pasic M Zipfel B Drews T Transapical placement of an uncovered aortic endostent for type A aortic dissection. *Circ Cardiovasc Interv* 2011; 4:e49–53. 22186109 10.1161/CIRCINTERVENTIONS.111.965178

[24] Abah U Dunne M Cook A Does quality of life improve in octogenarians following cardiac surgery? A systematic review. *BMJ Open* 2015; 5:e006904. 10.1136/bmjopen-2014-006904 PMC4420984 25922099

